# Development of the Inactivated QazCovid-in Vaccine: Protective Efficacy of the Vaccine in Syrian Hamsters

**DOI:** 10.3389/fmicb.2021.720437

**Published:** 2021-09-27

**Authors:** Kuandyk Zhugunissov, Kunsulu Zakarya, Berik Khairullin, Mukhit Orynbayev, Yergali Abduraimov, Markhabat Kassenov, Kulyaisan Sultankulova, Aslan Kerimbayev, Sergazy Nurabayev, Balzhan Myrzakhmetova, Aziz Nakhanov, Ainur Nurpeisova, Olga Chervyakova, Nurika Assanzhanova, Yerbol Burashev, Muratbay Mambetaliyev, Moldir Azanbekova, Syrym Kopeyev, Nurlan Kozhabergenov, Aisha Issabek, Moldir Tuyskanova, Lespek Kutumbetov

**Affiliations:** Research Institute for Biological Safety Problems, Gvardeiskiy, Kazakhstan

**Keywords:** inactivated vaccine, SARS-CoV-2, QazCovid-in, protective efficacy, Syrian hamsters

## Abstract

In March 2020, the first cases of the human coronavirus disease COVID-19 were registered in Kazakhstan. We isolated the SARS-CoV-2 virus from clinical materials from some of these patients. Subsequently, a whole virion inactivated candidate vaccine, QazCovid-in, was developed based on this virus. To develop the vaccine, a virus grown in Vero cell culture was used, which was inactivated with formaldehyde, purified, concentrated, sterilized by filtration, and then adsorbed on aluminum hydroxide gel particles. The formula virus and adjuvant in buffer saline solution were used as the vaccine. The safety and protective effectiveness of the developed vaccine were studied in Syrian hamsters. The results of the studies showed the absolute safety of the candidate vaccine in the Syrian hamsters. When studying the protective effectiveness, the developed vaccine with an immunizing dose of 5 μg/dose specific antigen protected animals from a wild homologous virus at a dose of 10^4^.^5^ TCID_50__/_mL. The candidate vaccine induced the formation of virus-neutralizing antibodies in vaccinated hamsters at titers of 3.3 ± 1.45 log2 to 7.25 ± 0.78 log2, and these antibodies were retained for 6 months (observation period) for the indicated titers. No viral replication was detected in vaccinated hamsters, protected against the development of acute pneumonia, and ensured 100% survival of the animals. Further, no replicative virus was isolated from the lungs of vaccinated animals. However, a virulent virus was isolated from the lungs of unvaccinated animals at relatively high titers, reaching 4.5 ± 0.7 log TCID_50_/mL. After challenge infection, 100% of unvaccinated hamsters showed clinical symptoms (stress state, passivity, tousled coat, decreased body temperature, and body weight, and the development of acute pneumonia), with 25 ± 5% dying. These findings pave the way for testing the candidate vaccine in clinical human trials.

## Introduction

Coronaviruses (CoV) are a large family of RNA-containing viruses that can infect humans and certain animal species (Weiss and Navas-Martin, 2005; To et al., 2013; Lau and Chan, 2015). In humans, coronaviruses can cause a number of diseases—from a mild form of acute respiratory infection to severe acute respiratory syndrome (Weiss and Navas-Martin, 2005; Su et al., 2016). Currently, the following coronaviruses are known to circulate among the population: HCoV-229E, HCoV-OC43, HCoV-NL63, HCoV-HKU1, severe acute respiratory syndrome (SARS)-CoV, and Middle East respiratory syndrome (MERS)-CoV, causing diseases of the upper respiratory tract and lungs with moderate severity (Lim et al., 2016; Ye et al., 2020). The new SARS-CoV-2 virus, which causes COVID-19, was added to this list and was registered in Wuhan, China at the end of December 2019 (Lu et al., 2020; Mackenzie and Smith, 2020). The new coronavirus infection has become a pandemic in a short time and has caused enormous socio-economic damage to the life and activities of humanity around the world. The rapid spread of the infection stopped many of the normal activities of entire states, and severe forms of the disease led to pneumonia-related death of many infected people (Lu et al., 2020). To prevent and combat the new coronavirus infection caused by the SARS-CoV-2 virus, many developed countries of the world quickly began to create means for specific prevention of the disease by developing various types of vaccines, including RNA vaccines, DNA vaccines, recombinant vector vaccines, subunit vaccines, inactivated vaccines, and live vaccines (Krammer, 2020; Poland et al., 2020; World Health Organization World Health Organization (WHO)., 2021). The advantages and disadvantages of these vaccines are detailed in a number of literature sources (Dong et al., 2020; Li et al., 2020).

According to the WHO (as of May 7, 2021), more than 236 potential vaccines are being developed worldwide, 63 of which are being tested in humans (World Health Organization World Health Organization (WHO)., 2021). Furthermore, 26 of these candidate vaccines are undergoing phase III clinical trials, some of which are already being used for mass vaccination in a number of countries (World Health Organization World Health Organization (WHO)., 2021).

Since the beginning of the pandemic, Kazakhstan has also started developing a domestic vaccine against the new coronavirus infection on five platforms, and one of these vaccines is based on the traditional technology of preparing an inactivated vaccine. This vaccine, under the name QazCovid-in, has successfully passed phase I and II clinical trials (Zakarya et al., 2021) and is in the second half of phase III trials.

In this article, we present the results of the creation of a new inactivated candidate vaccine QazCovid-in (or QazVac) and the study of its safety and immunological effectiveness in Syrian hamsters.

## Materials and Methods

### Virus

In the development of the QazCovid-in vaccine, the SARS-CoV-2/KZ_Almaty04.2020 strain (Kutumbetov et al., 2020) as deposited in the republican depository for the collection of microorganisms of the RSE “Research Institute for Biological Safety Problems” (RIBSP), Committee of Science of the Ministry of Education and Science of the Republic of Kazakhstan, was obtained from the SARS-CoV-2 virus isolated from a clinical sample of a patient with COVID-19. To prepare the vaccine and to infect hamsters, we used the 4th passage of the virus grown in a Vero cell culture at a titer of 7.25 ± 0.25 log TCID50/mL. The strain SARS-CoV-2/human/KAZ/KZ_Almaty/2020 was sequenced, and the complete genome sequence was deposited in GenBank under accession number MZ379258.1. The nucleotide sequence of the genome of strain SARS-CoV-2/human/KAZ/KZ_Almaty/2020 is 100% identical to that of the Wuhan-Hu-1 isolate (NC_045512.2). The results of the virus isolation from the clinical material are presented in Supplementary Material.

### Animals

Syrian hamsters (*n* = 100) of both sexes 3–4 months of age, weighing 100∼138 g, were randomly selected for experimentation. The hamsters were kept in individual ventilated Delta IVC-ZJ3 complexes (China).^1^ The animals were monitored daily by cataloging their general clinical condition, body temperature, body weight, appetite, and water intake. Concurrently, their food and water intake were assessed daily in accordance with their daily food intake (15 g dry food and 20 mL water per hamster). In addition, under normal physiological conditions, hamsters strategically store food in their oral cavity. However, under disease conditions (passive state, trembling, tousled coat), they do not store food in the mouth. Therefore, the appetite of the animals was assessed by considering both the amount of food and water left over from the daily food intake and the fluctuations in live weight.

### Preparation of the Candidate Vaccine

**FIGURE 1:**
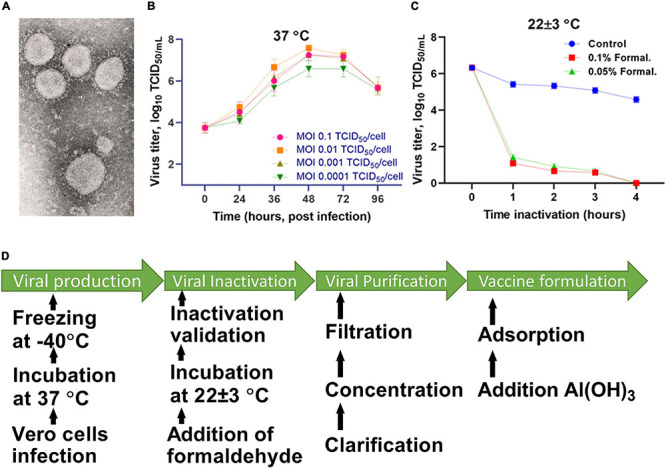
Scheme for preparing the candidate vaccine QazCovid-in. **(A)** Electronic micrograph of the SARS-CoV-2 virus (magnified by ×18,000, scale bar represents 100 nm). **(B)** The accumulation of the virus in Vero cells, depending on the multiplicity of the infecting dose (MOI). **(C)** The inactivation of the virus at 25°C. **(D)** The preparation steps for the QazCovid-in vaccine.

The virus was cultured in Vero cells (WHO, Lot No. CB0 or CB884) at 37°C for 48 h, and then inactivated with formaldehyde (Sigma Aldrich, St. Louis, MO, United States) for 24 h. The completeness of the inactivation of the virus was established by a bioassay in a cell culture carried out in three passages. The inactivated virus was purified and concentrated by a combined method using tangential flow ultra-filtration and exclusive chromatography. The purified and concentrated suspension of the inactivated virus was sterilized by membrane filtration (0.22 μm pore size). The purified virus, taken at a certain concentration of a specific protein in a phosphate-salt buffer, was mixed with an Al(OH)_3_ adjuvant (Alhydrogel^®^; InvivoGen, San Diego, CA, United States) and used as a candidate vaccine against SARS-CoV-2. The technology for preparing the candidate vaccine is schematically illustrated in Figure 1.

### Vaccine Safety Assessment

**FIGURE 2:**
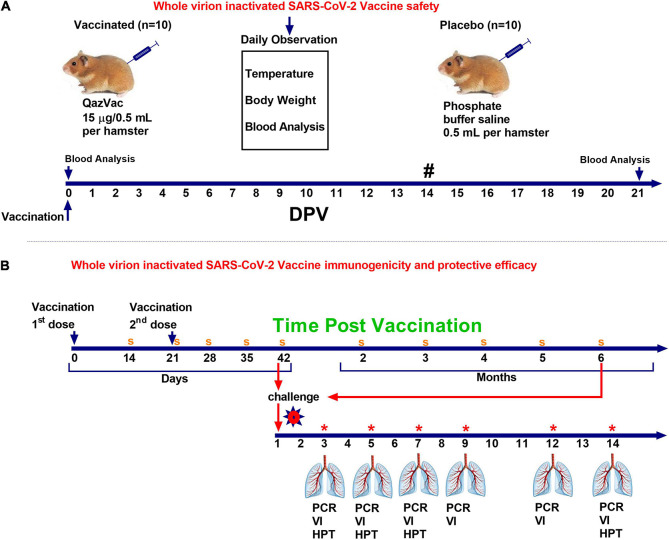
Study design of QazCovid-in vaccination in Syrian hamsters. **(A)** The study of the safety of the inactivated QazCovid-in vaccine in Syrian hamsters. **(B)** The determination of immunogenicity and evaluation of the protective efficacy of the inactivated QazCovid-in vaccine in Syrian hamsters. PCR, polymerase chain reaction; VI, virus isolation; HPT, histopathology. Notes (#)—measurement of body temperature and body weight was carried out within 14 days. (s)—sampling of blood serum to determine the titer of viurs-neutralizing antibodies (*)—on 3, 5, 7, 9, 12, and 14 DPI, samples of nasal and oral swabs were collected and examined by PCR and viral isolation in cell culture.

Syrian hamsters (*n* = 10) were administered the vaccine intramuscularly once at a dose of 0.5 mL, containing 15 μg of a specific virus (Spike) protein and 1.0 mg aluminum hydroxide in a phosphate-buffer saline solution (PBS). The control animals (*n* = 10) were injected with 0.5 mL PBS intramuscularly. The animals were monitored daily for 20 days, and their body temperature and live body weight were recorded. The day before vaccination and 21 days after the introduction of the vaccine, blood samples were taken from both groups and subjected to biochemical and hematological analyses. The scheme for evaluating the safety of the vaccine is shown in Figure 2A.

### Hematological and Biochemical Blood Tests

Hematological analysis of blood samples was performed using an automatic blood analyzer T-540 Coulter (Coulter Electronics, Hialeah, FL, United States). The concentration of hemoglobin and the counts of hematocrits, red blood cells, white blood cells, platelets, neutrophils, eosinophils, lymphocytes, and monocytes were determined. Biochemical studies of blood serum samples were carried out on VITALAB Selectra 2 in an automatic analyzer (Merck KGaA, Darmstadt, Germany) using commercial kits (DiaSys Diagnostic Systems GmbH, Holzheim, Germany), which determined the levels of total protein, total bilirubin, glucose, creatinine, aspartate aminotransferase, and alanine aminotransferase.

### Vaccine Immunogenicity Analysis and Protection Study

Hamsters (*n* = 60) were administered the vaccine twice at an interval of 21 day with an intramuscular dose of 0.5 mL containing 5 μg of a specific virus (Spike) protein adsorbed on aluminum hydroxide. The control group of animals (*n* = 20) was injected intramuscularly with 0.5 mL PBS. The animals were monitored daily, with body temperature measurement and live weight determined using electronic scales for 42 days. Blood samples were taken from the animals before vaccination for the determination of virus neutralizing antibodies (VNA) to the SARS-CoV-2 virus. Then, on the 14th, 21st, 28th, 35th, 42nd, 60th, 90th, 120th, 150th, and 180th day post vaccination (DPV), blood samples were collected from all vaccinated animals to determine the dynamics of the formation of VNA in a neutralization test.

To establish the protective efficacy of the vaccine, the infectious process was modeled on vaccinated and control (unvaccinated) Syrian hamsters by injecting them with the wild homologous SARS-CoV-2 virus at a dose of 10^4^.^5^ TCID_50_/animal in a volume of 100 μL intranasally. To do this, half each of the number of the vaccinated and unvaccinated Syrian hamsters were exposed to a virulent virus 42 days after the first vaccination, and the remaining half of vaccinated and unvaccinated animals was exposed to a virulent virus after 6 months.

After the challenge with a virulent virus, the animals were monitored daily, recording their general condition, appetite, body temperature, live weight, and signs of pathologies. To compare the changes with those in unvaccinated and uninfected healthy hamsters, 18 hamsters were kept in individual cages and compared with experimental hamsters at 3, 5, 7, 9, 12, and 14 DPI. On days 3, 5, 7, 9, 12, and 14, samples of oral and nasal swabs were collected to determine the presence of a virulent pathogen.

To evaluate the effectiveness of the candidate vaccine via intramuscular administration of animals, 3 (*n* = 5), 5 (*n* = 5), 7 (*n* = 5), 9 (*n* = 5), 12 (*n* = 5), and 14 (*n* = 5) days after the challenge infection, the animals were euthanized by CO_2_ inhalation using a standard two-cell kit AE0904. The gas consumption was 3.5 L/min per cell for 2–4 min. The onset of animal death was monitored by the absence of respiration and fading of the eyes of each animal, after which a visual examination and autopsy were performed, and the lungs were selected for virus isolation and histological examination. The design of the study is shown in Figure 2B.

### Histological Examination of the Lungs

For microscopic analysis, lung tissue samples were taken from all the studied animals and fixed in a 10% solution of neutral formalin. The tissue pieces were left in formalin overnight at 25°C, then treated according to the standard histological technique procedure (dehydration, clearing, and compaction). Tissue sections with a thickness of 4–5 μm were prepared from paraffin blocks using a sled microtome. For a general overview, the histological sections were stained with hematoxylin and eosin. Microscopic analysis and photography were carried out under a Nikon ECLIPSE 50i microscope equipped with a Nikon Digital Sight DS-Fi1 camera (Tokyo, Japan).

### Neutralizing Assay

Sera from blood samples collected from immunized animals were inactivated at 56°C for 0.5 h and serially diluted with cell culture medium in twofold steps. The diluted sera were mixed with a virus suspension of 100 TCID_50_ in 96-well plates at a ratio of 1:1, followed by 2 h incubation at 37°C in a 5% CO_2_ incubator. Vero cells (1–2 × 10^4^) were then added to the serum-virus mixture, and the plates were incubated for 5 days at 37°C in a 5% CO_2_ incubator. The cytopathic effect (CPE) of each well was determined by microscopy, and the neutralizing antibody titer was calculated by the dilution number of 50% protective condition in accordance with Reed and Muench (1938).

### Virus RNA Isolation

The virus RNA was extracted from clinical samples using the QIAamp viral RNA mini kit (Qiagen, Hilden, Germany) according to the manufacturer’s instructions.

### Viral RNA Analysis

SARS-CoV-2 RNA was assessed by RT-PCR using an approach similar to that previously described (Corman et al., 2020; Wölfel et al., 2020). The following primers and probe were used to amplify the N gene of the SARS-CoV-2 virus: N_Sarbeco_F (CACATTGGCACCCGCAATC), N_Sarbeco_R (GAGGAACGAGAAGAGGCTTG), and N_Sarbeco_P (FAM-ACTTCCTCAAGGAACAACATTGCCA-BBQ) (Corman et al., 2020). The viral genome was evaluated by quantitative real-time PCR using the Superscript^®^ III Platinum One-Step RT-PCR system kit with the Platinum^TM^ Taq DNA polymerase system (Invitrogen, Waltham, MA, United States) according to the manufacturer’s instructions. The reactions were carried out in a thermal cycler of the Rotor-Gene 6000 series (Qiagen) with the following program: 1 reverse transcription cycle at 50°C for 20 min., 1 cycle of 95°C for 3 min, followed by 45 cycles of 95°C for 15 s and 58°C for 30 s.

### Isolation of the Virus in Cell Culture

The virus was isolated from lung samples in which viral RNA was detected by PCR. For this purpose, a 20% organ-tissue suspension was prepared from the lungs of hamsters using a generally accepted technique. Before infection, all cell culture flasks were microscopically examined and only cultures with a good, typical monolayer were selected. After removing the culture medium, 0.5 mL of the prepared 20% suspension was applied to the monolayer of Vero cell culture and kept for 60 min at 37°C. Thereafter, the inoculate was removed, the monolayer was washed three times with PBS solution, DMEM maintenance medium was added with fetal blood serum, and cultivation was continued at 37°C with daily microscopy of the cell culture monolayer. The presence of the virus was determined by the CPE in infected cell cultures compared to that in the uninfected control cell culture. In the absence of CPE in a Vero cell culture infected with biomaterial samples, “blind” passaging was performed for at least three replicates.

### Facility and Ethics Statements

Challenge experiments in animals and all other experiments with live SARS-CoV-2 virus were performed under ABSL-3 conditions and BSL-3 facilities in the RIBSP. This study was performed in compliance with national and international laws and guidelines on animal handling, and the experimental protocol was approved by the Committee on the Ethics of Animal Experiments of the RIBSP of the Science Committee of the Ministry of Education and Science of the Republic of Kazakhstan (permit numbers: KZ0520/013, KZ1120/014).

### Statistics

Statistical analysis was performed using Prism 8.4.2 (GraphPad, San Diego, CA, United States) for vaccinated and control groups followed by the Student’s parametric test, ANOVA, and the Wilcoxon-Mann-Whitney non-parametric test. One of the criteria for evaluating the effectiveness was the percentage of survival (PV). PV was evaluated by the Kaplan-Meyer method, and PV indicators in the groups of vaccinated and unvaccinated animals were compared by the log-rank method. *p* < 0.05 was considered statistically significant.

## Results

### Safety of the QazCovid-in Vaccine Candidate in Hamsters

#### Clinical Monitoring

**FIGURE 3:**
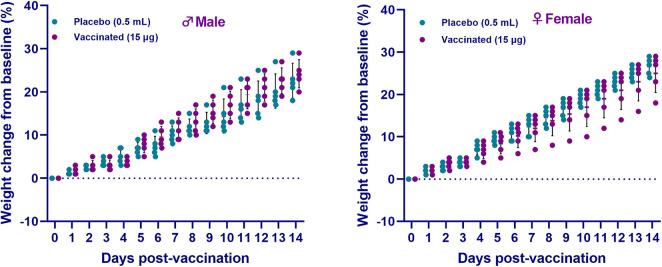
The changes in the live weight of hamsters after administration of the QazCovid-in vaccine candidate. The vaccinated groups were compared to the placebo group (PBS). Data are presented as mean ± standard deviation (SD). The statistical significance was assessed using two tailed ANOVA between the two groups with a Šídák’s multiple comparisons test; *p* < 0.05 was considered statistically significant.

During the entire observation period, the general condition of the animals remained satisfactory (free and active movement in cages, healthy food and water intake, no observed pathologies). After vaccine administration, measurement of the live weight of hamsters showed an increase in weight in both sexes of hamsters in all groups for the entire observation period (Figure 3). Concurrently, there was no significant difference between the vaccinated group and the placebo (*p* ≥ 0.05). Furthermore, no changes in the growth and development of the animals were detected. The temperature response remained within the physiological limit. The obtained data of clinical observation indicated that the candidate vaccine, when administered intramuscularly at a dose of 15 μg/0.5 mL/animal, did not have a negative effect on the overall clinical condition (behavior, appetite, etc.) of the test animals during the entire observation period. None of the 10 animals vaccinated with an excessive dose of the vaccine showed any signs of the disease during the entire period of clinical observation. The data obtained showed the absence of local and irritant effects of the vaccine, as well as the ability of the drug to induce allergic reactions of immediate and delayed types, which minimizes the risks of anaphylactic reactions (anaphylactic shock, edema, etc.) after vaccine administration.

#### Hematological and Biochemical Studies

Hematological and biochemical blood tests showed that all the studied parameters remained within physiological limits during the entire follow-up period (Tables 1, 2). The hematological and biochemical blood parameters after immunization did not significantly differ from the baseline established before vaccination in all groups (*p* ≥ 0.05).

**TABLE 1:** Average values (M ± SD) of hematological blood parameters of vaccinated hamsters after administration of the QazCovid-in vaccine candidate.

**Parameters**	**Animal groups (*n* = 20)**			
	**Placebo**		**Vaccinated**	
	**M (*n* = 5)**	**F (*n* = 5)**	**M (*n* = 5)**	**F (*n* = 5)**
RBC (× 10^6^ cells/μL)	7.7 ± 1.8	8.0 ± 2.1	7.6 ± 1.1	7.8 ± 1.1
WBC (× 10^9^ cells/μL)	7.6 ± 2.7	7.7 ± 2.3	7.2 ± 3.0	8.3 ± 4.3
Plat (× 10^9^ cells/μL)	37.0 ± 12.5	40.1 ± 11.4	37.3 ± 9.1	43.1 ± 8.7
Hb (g/dL)	15.9 ± 2.3	14.8 ± 1.7	16.4 ± 2.5	14.4 ± 2.8
Hct	45.7 ± 6.1	44.9 ± 8.2	42.6 ± 9.1	41.3 ± 8.1
MCH	59.6 ± 8.1	58.4 ± 11.2	60.5 ± 13.2	60.1 ± 10.2
MCHC	34.8 ± 5.7	36.8 ± 5.4	35.9 ± 7.1	35.9 ± 7.1
Neut (× 10^9^ cells/μL)	1.7 ± 1.1	1.7 ± 1.1	1.8 ± 1.3	1.8 ± 1.9
Mono (× 10^9^ cells/μL)	0.9 ± 0.9	0.9 ± 0.9	1.0 ± 0.7	1.1 ± 0.9
Lymp (× 10^9^ cells/μL)	4.6 ± 2.7	4.6 ± 2.7	6.3 ± 2.7	5.8 ± 2.9
Eos (× 10^9^ cells/μL)	0.4 ± 2.2	0.3 ± 2.2	0.3 ± 2.9	0.4 ± 2.9
Baso (× 10^9^ cells/μL)	0.02 ± 0.40	0.02 ± 0.20	0.03 ± 0.90	0.03 ± 0.80

*M, male; F, female; RBC, red blood cells; WBC, white blood cells; Plat, platelets; Hb, hemoglobin; Hct, hematocrit; MCH, mean cell hemoglobin; MCHC, mean cell hemoglobin concentration; Neut, neutrophils; Mono, monocytes; Lymp, lymphocytes; Eos, eosinophils; Baso, basophils.*

**TABLE 2:** Average values (M ± SD) of the main biochemical parameters of hamster blood serum in determining the safety of the test drug.

**Parameters**	**Animal groups (*n* = 20)**			
	**Placebo**		**Vaccinated**	
	**M (*n* = 5)**	**F (*n* = 5)**	**M (*n* = 5)**	**F (*n* = 5)**
Total protein (mg/dL)	79.5 ± 8.8	79.4 ± 6.9	78.3 ± 7.8	81.4 ± 7.6
Glucose (mg/dL)	8.0 ± 1.4	7.9 ± 0.3	8.0 ± 0.9	8.0 ± 1.8
Ñreatinine (mg/dL)	66.9 ± 3.8	66.7 ± 3.9	67.0 ± 3.2	67.3 ± 3.7
Total bilirubin (mg/dL)	9.2 ± 1.1	8.6 ± 1.7	7.8 ± 0.6	7.9 ± 0.7
AST (U/L)	97.8 ± 25.2	93.5 ± 14.8	97.9 ± 22.3	94.1 ± 23.0
ALT (U/L)	71.4 ± 8.9	72.1 ± 11.2	72.5 ± 8.2	71.1 ± 11.2
Urea (mg/dL)	4.9 ± 0.9	4.8 ± 0.8	4.8 ± 0.2	4.9 ± 0.3

*M, male; F, female; AST, aspartate transaminase; ALT, alanine aminotransferase.*

### Antibody Responses After QazCovid-in Vaccination

**FIGURE 4:**
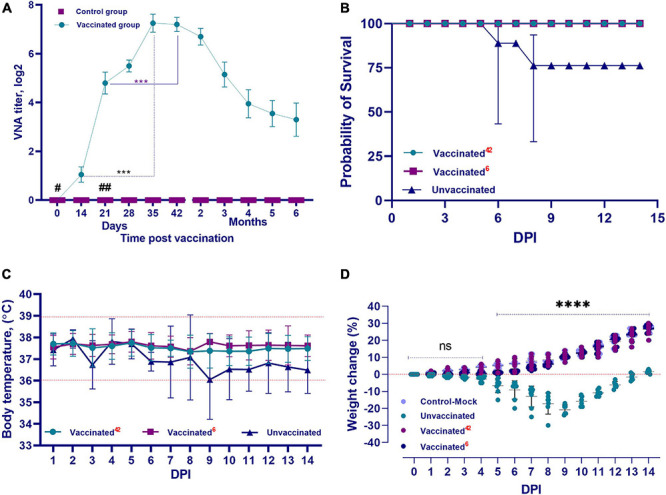
Performance indicators of the inactivated QazCovid-in. **(A)** Vaccine- virus neutralizing antibodies (VNA) in blood serum samples of Syrian hamsters before (day 0) and after vaccination. The graph shows the average values of VNA titers with standard deviation (mean ± SD). ^∗∗∗^*p* < 0.001. **(B)** The survival rate of vaccinated and unvaccinated hamsters, after infection with the wild SARS-CoV-2 virus. **(C)** The temperature reaction of vaccinated and unvaccinated hamsters after challenge with wild homologous SARS-CoV-2 virus. There was no significant difference in average body temperature between the vaccinated and unvaccinated groups (*p* ≥ 0.05). **(D)** Changes in the body weight percentage of vaccinated and unvaccinated hamsters after challenge with the wild homologous SARS-CoV-2 virus; 42 indicates the vaccinated group challenged 42 days after vaccination, 6 indicates the vaccinated group challenged 6 months after vaccination. ns indicates no significance. ^#^The first dose was inoculated on day 0. ^##^The second dose was administrated on the 21st day.

The QazCovid-in vaccine administered to the vaccinated hamsters pronouncedly stimulated the formation of humoral immunity factors (Figure 4A). After the first dose of the vaccine was administered on day 0, VNA was detected in animal serum samples on day 14 at a titer of 1.0 ± 0.5 (CI 95%, < 0.5–2.0) log_2_. On day 21, the titers of these antibodies increased to 4.8 ± 0.9 (CI 95%, < 3.0–6.0) log_2_. On the 28th day after the first dose of the vaccine, the level of antibodies increased slightly [CI 95%, 5.5 ± 0.5 log_2_ (CI 95%, < 5.0–7.0)], and on the 35th day after the first dose, their titer reached the highest values [7.25 ± 0.78 log_2_ (CI 95%, < 7.0–8.0)], and remained at this level until the 42nd day. A decrease in the VNA titer was observed at 2 months after the first dose [6.7 ± 0.7 log_2_ (CI 95%, < 6.0–8.0)], and at 6 months, the antibody titer averaged 3.3 ± 1.45 (CI 95%, < 2.0–7.0) log_2_. Furthermore, there was a significant difference in the level of VNA in the blood serum samples of immunized hamsters between the first and second doses (assessed between 14 and 35 DPV and between 21 and 42 DPV, both with *p* ≤ 0.001).

### Protective Efficacy of QazCovid-in Vaccine Candidates

#### Clinical Monitoring During Challenge Study on Day 42 Post Vaccination

After the challenge infection, the vaccinated group of hamsters completely lacked any signs of the disease, and all vaccinated animals remained alive and clinically healthy for 14 days. Their body temperature fluctuated within the physiological limit (36.0–39.0°C) (Figure 4C). The vaccinated hamsters gained weight of within 2–3% of the total initial weight from 2 to 5 days after infection. From 6 to 14 days, they began to actively gain weight in the range of 10–30% of the total initial weight (Figure 4D).

#### Clinical Monitoring During Challenge Study on Month 6 Post Vaccination

In the first 5–6 days after the challenge infection with the virulent virus, there was no increase in the live weight of the vaccinated hamsters, but the live weight began to increase from day 7 until the end of the observation. The live weight of the vaccinated hamsters at the end of the observation did not differ from that of the control group (unvaccinated and uninfected) (Figure 4D). In vaccinated animals, there were no cases of disease and death after the challenge with the SARS-CoV-2 virus (Figure 4B).

#### Clinical Monitoring of Unvaccinated Hamsters After Challenge Infection

Among the hamsters of the unvaccinated control group after challenge infection with the virulent virus, in addition to the complete absence of weight gain during the entire observation period, there was a decrease in live weight by 20–30% compared to the initial weight (Figure 4D). The survivors were only able to regain their initial weight after 14 days.

Concurrently, in the animals of the unvaccinated group, there was a stress state, passivity, ruffled hair, and stroking of the nasal mirror with the limbs, which was a sign of itching. Starting from the 3rd DPI, some unvaccinated animals showed a decrease in body temperature to 34.5°C (Figure 4C), as well as a decrease in live weight (Figure 4D); however, there were no significant differences between temperatures of the groups (*p* ≥ 0.05). Hypothermia in animals was recorded from 3 to 8 days after inoculation of the virus, and in such cases, the outcome of the pathology was fatal within the same or next day. The peak of the disease occurred on 5–8 DPI. The death of unvaccinated animals occurred on the 3rd DPI. Concurrently, two hamsters died on the 3rd DPI, two more hamsters on the 4th day, and one hamster died on the 6th and 8th days, respectively. Mortality in the control group averaged 30%, with 100% morbidity (Figure 4B).

### Determination of Viral RNA From Nasal and Oral Swabs From Vaccinated and Unvaccinated Animals After Challenge Infection

#### Group Infected on Day 42 Post Vaccination

**FIGURE 5:**
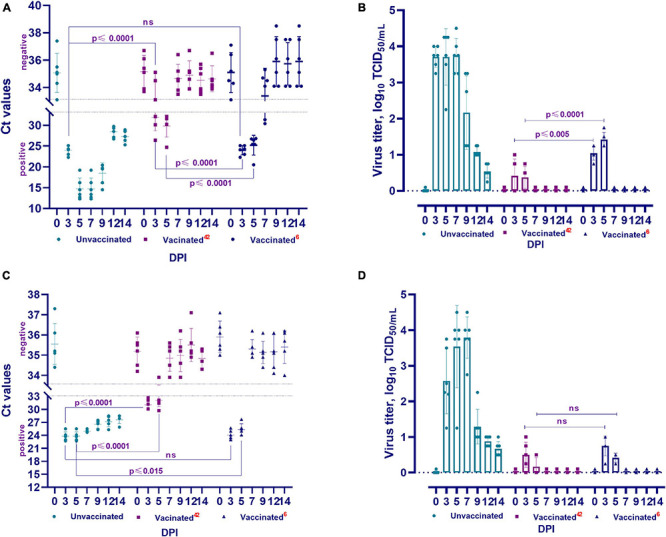
Viral RNA load and isolation for cell culture of challenged animals. The PCR results of nasal swabs **(A)** and oral swabs **(C)**, and the virus isolated for cell culture from clinical samples from vaccinated and unvaccinated hamsters after challenge with wild homologous SARS-CoV-2 virus (**B:** nasal swabs; **D:** oral swabs). Ct values between 10 and 33 are positive, while Ct values ≥ 33.1 are negative; 42 indicates the vaccinated group challenged at 42 days after vaccination, 6 indicates the vaccinated group challenged 6 months after vaccination. ns indicates no significance.

The viral RNA was detected in the nasal and oral swabs of vaccinated hamsters 3 and 5 days after the challenge. No viral RNA was found at 7, 9, 12, and 14 days (Figures 5A,C). It should be noted that viral RNA was detected in three (Ct < 26.4–32.1) of the 6 tested samples (nasal swabs on day 3), while the remaining 3 samples were negative for viral RNA (Ct < 33.1–35.1). The viral RNA was detected in all oral swab samples within 3 days (Ct < 29.7–32.1). Only one (Ct < 33.9) of all tested oral swabs tested negative for viral RNA 5 days after the challenge infection.

#### Group Infected on Month 6 Post Vaccination

RNA of the virus was also detected in nasal swabs (Ct < 22.3–31.3) collected from days 3 to 7 and in oral swabs (Ct < 23.0–27.7) collected from days 3 to 5 (Figures 5A,C). The remaining samples collected from the oral and nasal cavities up to the 14th day showed negative results (Ct < 33.3–37.5). It should be noted that the Ct values of positive samples at 3 and 5 DPI were between 20.1 and 26.5 compared to those at 42 DPV, and viral RNA was detected in 2 samples at 7 DPI (Ct < 30.4–31.3).

#### Group Unvaccinated

In all samples (oral and nasal swabs) obtained from unvaccinated animals, SARS-CoV-2 virus RNA was detected (Ct < 12.3–29.7) at all studied time points after the challenge infection.

### Isolation of the Virus in Cell Culture From Nasal and Oral Swabs From Vaccinated and Unvaccinated Animals After Challenge Infection

#### Group Infected on Day 42 Post Vaccination

The viral titer was 0.75 − 1.0 log TCID_50_/mL in three of five samples collected from the nasal cavity of vaccinated hamsters at 3 DPI. The virus was isolated in four of five samples at 5 DPI, but the viral titer decreased (0.50 − 0.75 log TCID_50_/mL) (Figure 5B). At 3 DPI, the virus was detected in four of five samples collected from the oral cavity, and the viral titer was 0.50 − 1.0 log TCID_50_/mL. At 5 DPI, the virus was isolated in 2 samples and its titer was 0.50 log TCID_50_/mL (Figure 5D). Between the 7th and 14th days, the virus was not isolated from samples taken from the nasal and oral cavities (Figures 5B,D).

#### Group Infected on Month 6 Post Vaccination

The virus was isolated from four of five samples collected from the nasal cavity on the 3rd DPI and its titer ranged from 0.75 to 1.25 log TCID_50_/mL. The titer of the virus in all samples was significantly higher at 5 DPI (1.25 − 1.75 log TCID_50_/mL) than at 3 DPI (Figure 5B). In oral samples, the titer of the virus on the 3rd DPI was 0.75 − 1.0 log TCID_50_/mL, but it was significantly lower (0.25 − 0.50 log TCID_50_/mL) on the 5th DPI (Figure 5D). It should be noted that at 42 DPV and 6 months after vaccination, there was a significant difference between the titers of the virus released from the nasal cavity (from *p* ≤ 0.005 to *p* ≤ 0.0001), but there was no significant difference (*p* ≥ 0.05) between the titers of the virus released from the oral cavity (Figures 5B,D).

#### Group Unvaccinated

The virus was isolated from all nasal and oral samples collected from unvaccinated animals from the 3rd to 14th DPI, with a titer of 0.54 ± 0.10–3.79 ± 0.23 log TCID_50_/mL. In studies of vaccinated animals, this pathogen was isolated from samples collected from the 3rd to 5th DPI, with a significantly lower titer than that of the virus isolated from unvaccinated animals (Figures 5B,D). Concurrently, the difference between the titers of the virus isolated in the two groups of animals was significant (from *p* ≤ 0.002 to *p* ≤ 0.0001).

### Pathology and Viral Load in Lung Tissue After Challenge Infection

#### Group Infected on Day 42 Post Vaccination

**FIGURE 6:**
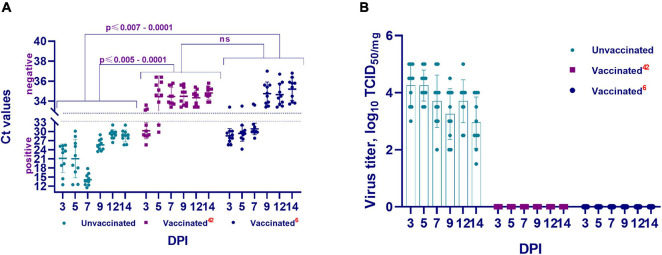
The presence and concentration of virulent virus in the lungs of vaccinated and unvaccinated hamsters. This figure shows data from the 3rd, 5th, 7th, 9th, 12th, and 14th DPI with the wild homologous SARS-CoV-2 virus, specifically the genomic RNA in the lungs **(A)**, and virus isolation from the lungs in Vero cells. **(B)** Ct values between 10 and 33 are positive, while Ct values ≥ 33.1 are negative; 42 indicates the vaccinated group at 42 days after vaccination; 6 indicates the vaccinated group challenged 6 months after vaccination; ns indicates no significance; DPI, days post infection.

There were no visible pathologies in the thoracic and abdominal organs of the euthanized hamsters on the 3rd, 5th, 7th, 9th, 12th, and 14th DPI of the vaccinated hamsters. Further, on the 3rd DPI, PCR examination of the lung tissue revealed viral RNA in three of 10 hamsters (Ct < 25.6–28.9), and in two of 10 hamsters on the 5th DPI (Ct < 29.8–32.1). However, full clearance of viral RNA was observed between 7 and 14 DPI (Ct < 33.4–35.7) (Figure 6A). This was confirmed when all samples were examined in a cell culture wherein no virus was isolated (Figure 6B).

#### Group Infected on Month 6 Post Vaccination

In this group also, there were no visible pathological changes on examination of the thoracic and abdominal cavities. However, viral RNA was detected in lung tissue from 6 test sites at 3, 5, and 7 DPI (Ct < 24.2–32.4). Full clearance of viral RNA was observed between 9 and 14 DPI (Ct < 33.4–37.0) (Figure 6A). This was also confirmed by cell culture wherein no virus was isolated (Figure 6B).

#### Group Unvaccinated

In the abdominal cavity organs of this group, no visible pathologies were detected. However, opening of the thoracic cavity in unvaccinated hamsters, uneven staining, and spot hemorrhages in the lungs were observed. The bronchial and mediastinal lymph nodes were enlarged and swollen.

Further, the presence or absence of the viral genome in the upper and lower respiratory tracts of unvaccinated animals in the abovementioned periods after challenge infection was established by PCR (Figure 6). It was revealed that the genome of the SARS-CoV-2 virus was present in the upper and lower respiratory tracts of unvaccinated hamsters at the studied time (Figure 6A). Concurrently, the concentration of the virus genome in the vaccinated group of hamsters (Ct 24.20–36.80) was significantly lower (from *p* ≤ 0.005 to *p* ≤ 0.0001) than that in the unvaccinated group of animals (Ct 11.53–30.10).

Cell culture conducted to detect the presence of a replicative virus in the respiratory tract tissue samples obtained from unvaccinated animals yielded positive results, i.e., in unvaccinated animals, the virulent virus in the lung tissues was detected at relatively high titers, reaching 4.5 ± 0.7 log TCID_50__/_mL (Figure 6B).

### Histopathology of Lung Tissue From Vaccinated and Unvaccinated Syrian Hamsters After Challenge Infection

#### Histopathological Changes in Lung Tissues of the Unvaccinated Hamsters After Challenge Infection

**FIGURE 7:**
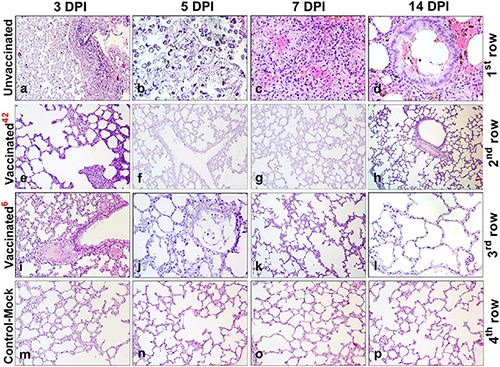
Paraffin sections of the lungs of vaccinated and unvaccinated hamsters. Pathohistological picture of the lung of unvaccinated hamsters (**a–d**, 1st row) and vaccinated hamsters challenged after 42 days (**e–h**, 2nd row), and vaccinated hamsters challenged after 6 months (**i–l**, 3rd row) on days 3, 5, 7, and 14, respectively after challenge with the wild homologous SARS-CoV-2 virus; 42 indicates vaccinated group challenged 42 days after vaccination; 6 indicates vaccinated group challenged 6 months after vaccination. The 4th row shows the histological picture of the lung of control hamsters on days 3, 5, 7, and 14 **(m–p)**, indicative of normal lung microstructure. Alveoli, bronchioles, capillaries, and the respiratory membrane are clearly observed. The images were stained with hematoxylin and eosin. Image magnification: × 400. DPI, days post infection.

Histological analysis after inoculation with a virulent virus in the lungs of animals of the unvaccinated group revealed chronic interstitial inflammatory cells during all the study periods (Figures 7a–d). On the 3rd DPI, pathohistological changes in the lung were characteristic of injuries in the initial exudative phase of acute respiratory distress syndrome (Figure 7a). There was extensive diffuse alveolar damage, atrophy, and collapse of the alveoli, desquamation of atypical pneumocytes, and peribronchial focal accumulation of pulmonary-associated lymphoid tissue. On the 5th DPI, characteristic atypical cells were detected with pale colored nuclei of different sizes, fibrin exudate, and diffusely localized lymphocytes (Figure 7b). On the 7th DPI, the lungs of unvaccinated hamsters showed hemorrhagic necrosis, microthrombi, atypical cells and multinucleated giant syncytial cells, multiple inflammatory cells, and fragments of apoptosis (Figure 7c. Furthermore, the dominant part of the microstructural elements of the lung parenchyma had already been restored. We also found the bronchioles in a state of recovery with an internal content of red blood cells, microthrombs, hemorrhagic necrosis, and diffuse infiltration of lymphoid tissue (Figure 7d).

#### Histopathological Changes in Lung Tissues of the Vaccinated Hamsters After Challenge Infection at 42 DaysPost Vaccination

After challenge, no pathological changes were found in the lungs of vaccinated hamsters. The microstructures of the lungs were preserved throughout the observation period (Figures 7e–h).

#### Histopathological Changes in Lung Tissues of the Vaccinated Hamsters After Challenge Infection at 6 Months Post Vaccination

Histopathology of the lungs of animals of the vaccinated group was limited to minor alveolar changes, which were detected only on the 3rd and 5th DPI with the virus, and were less noticeable compared to those in the unvaccinated animals (Figures 7i–l). On the 3rd DPI, the lungs were in an inflammatory state (Figure 7i). A small amount of exudate was detected in the lumen of some alveoli. However, on the 5th DPI (Figure 7j) in the lung, there was an expansion of the zones of diffuse damage to the alveoli. Nevertheless, a significant part of the organ still functioned normally, i.e., it had a characteristic microstructure. The dominant part of the lung parenchyma had a normal microstructure on the 7th DPI (Figure 7k). Furthermore, the connective tissue bases (layers) of the walls of the bronchioles and alveoli had been restored (Figure 7k). On the 14th DPI, the hamster’s lungs were externally covered with pleura formed from the mesothelium and dense elastic connective tissue (which partitions the lungs into lobes and lobules). The bronchioles of the lungs are covered with a ciliated prismatic or cubic single-layer epithelium (Figure 7l). On the 7th and 14th DPI, no changes in the lungs were observed in the animals of the vaccinated group (Figures 7j–l). It should be noted that there were no pathological changes in the lung tissues of control-mock hamsters (Figures 7m–p).

## Discussion

Due to the COVID-19 pandemic, a number of countries have not only developed their own vaccines over the past 1–1.5 years, but also successfully conducted three phases of clinical trials (Polack et al., 2020; Baden et al., 2021; Logunov et al., 2021; Voysey et al., 2021).

In Kazakhstan, research into developing a vaccine against the new coronavirus infection begun within the framework of a state-sponsored special scientific and technical program when the first cases of the disease were detected in the country in March 2020. On this basis, the SARS-CoV-2 virus was isolated from a clinical sample of a sick patient. The research program included the development of five different vaccines: an inactivated whole-virion vaccine based on a virulent virus, a subunit protein vaccine, two recombinant vaccines based on influenza and capripoxvirus vectors, and a live/replicative vaccine based on an attenuated homologous virus.

Initially, we mostly focused on the development of an inactivated vaccine. First, the technological basis for the preparation of such a vaccine is more conventional in virological practice, and has proven effectiveness in ensuring biological safety in the fight against dangerous human diseases such as influenza and polio (Krishnan et al., 1983; Katayose et al., 2011; Grassly, 2014; Klein et al., 2020). Second, inactivated vaccines, due to the use of inactivated virus, guarantee greater safety. Third, due to the use of a virus containing the entire complex of specific antigens in the composition of the whole-virion biomass, this vaccine type provides sufficient immunological efficacy.

Inactivated vaccines against COVID-19 coronavirus infection are being developed in several countries, including Russia and China. Evaluating the results of a trial in Brazil, China recently reported a low efficacy rate (50%) of its inactivated COVID-19 vaccine (Faria et al., 2021). The decrease in the effectiveness of this vaccine, in contrast to the information previously published about a sufficiently high immunogenicity in China (Gao et al., 2020; Yao et al., 2021), may either be associated with: (1) the manifestation of a mutated version of the virus (Garcia-Beltran et al., 2021; Wang et al., 2021), (2) excessive denaturation of the virus epitope used in the vaccine during the disturbed inactivation regime, or (3) other factors that may have a negative impact on the immunological effectiveness of the vaccine during production. The negative effect of formaldehyde on the antigenic structure of viral pathogens has been confirmed by experimental data of some studies (Metz et al., 2004). For example, when treated with this chemical, the antigenic properties change and the immunogenicity of these vaccines against, i.e., viral hepatitis A and B, polio, bovine herpes virus type 1, and influenza decreases when tested on mouse models (Peterson et al., 1984; Duque et al., 1989; Tano et al., 2007; Furuya et al., 2010; Wilton et al., 2014). Considering these facts, to maximize the preservation of antigenicity, we used the most sparing concentration of the chemical inactivant and the temperature-time mode of inactivation of the virus. As a result, the SARS-CoV-2 virus, inactivated with formaldehyde in the selected mode, retained its morphological and structural integrity, which was confirmed by electron microscopy, and stimulated the formation of specific antibodies in the experimental animal model, which neutralize the virulent virus equally as post-infectious antibodies.

In research and production technologies, beta-propiolactone and gamma rays are also used as inactivants in the inactivation of pathogens of viral diseases (Delrue et al., 2012). Further, researchers note that under the influence of beta-propiolactone, specific proteins of pathogens undergo significant modification (Delrue et al., 2012). The use of gamma radiation to inactivate viruses is more optimal, since it prevents the formation of free radicals that induce toxicity and reduces the risk of possible changes in the structure of the viral protein. However, there is no detailed information about the use of gamma rays in the preparation of a vaccine against SARS-CoV-2, except in isolated cases (Karakus et al., 2021). Therefore, we did not use this method of virus inactivation in our studies.

According to the literature, hamsters have unique physiological characteristics, making them suitable as an experimental model for biomedical research (Dutta and Sengupta, 2019), including for viral diseases. It was found that the SARS-CoV virus replicates in these animals (Roberts et al., 2005, 2008), and due to their susceptibility to SARS-CoV-2 infection (Chan et al., 2020; Imai et al., 2020; Sia et al., 2020), they are recommended as a biological model for COVID-19. Therefore, we assessed the safety of the tested vaccine on Syrian hamsters. There were no visible post-vaccination clinical reactions in all hamsters. The clinical data, hematological data, and biochemical parameters measured using blood serum were used as safety indicators of the safety of the tested vaccine. To assess the safety of inactivated vaccines against COVID-19 in preclinical trials, the main focus has been to detect pathological changes by pathomorphological and histological methods in parenchymal organs, as well as to perform hematological and biochemical analyses of blood (Gao et al., 2020; Karakus et al., 2021; Kandeil et al., 2021; Yao et al., 2021). We did not conduct pathomorphological and histological studies of parenchymal organs when assessing the safety of the vaccine. Results of hematological and biochemical blood tests showed no changes in blood parameters of vaccinated hamsters when comparing with those in the placebo group. In animals of the vaccinated group, the increase in live weight was higher than that in the control group (placebo), which indicated the absence of toxicity of the tested vaccine.

The main indicators of the immunogenic effectiveness of vaccine preparations are humoral immunity in the form of specific antibodies and the resistance of vaccinated animals to the virulent pathogen. Evaluation of the immunity of vaccinated hamsters, conducted by the presence and titer of specific VNAs, showed that the QazCovid-in vaccine stimulates the formation of VNAs in animals at titers up to 6–8 log2, which were detected during the next 6 months (follow-up period) after double immunization with an interval of 21 days. The formation of humoral immunity factors in model animals and humans when using vaccines against COVID-19 has been reported (Gao et al., 2020; Kandeil et al., 2021; Mohandas et al., 2021). These factors are important indicators in assessing the immunogenic reactivity of the vaccinated organism and the immunogenic effectiveness of the vaccine preparation used. However, the most reliable way to assess the intensity of immunity formed in response to vaccination is to determine the body’s resistance to disease when infected with a virulent pathogen. Therefore, a number of researchers have shown the protection of model animals immunized with the tested vaccines from coronavirus infection caused by SARS-CoV and SARS-CoV-2. Concurrently, the authors evaluated the effectiveness of their vaccine against SARS-CoV and SARS-CoV-2 (Roberts et al., 2010; Karakus et al., 2021; Kandeil et al., 2021; Mohandas et al., 2021) based on the levels of titers, isolated indicator virulent virus, and histopathological changes developing in the lungs of animal models. To assess the immunogenic effectiveness of the QazCovid-in vaccine in this study, we used these previously published indicators, along with data on the dynamics of body weight gain, body temperature, clinical indicators, and the outcome of the disease in a comparative order in the experimental and control groups of animals.

However, detailed information about the pathogenesis and deaths among hamsters used to study infection with the virulent SARS-CoV-2 virus has not been reported. According to related studies (Chan et al., 2020; Imai et al., 2020; Sia et al., 2020), model animals of this species in all cases survived, despite the use of high doses of the pathogen for inoculation. In all studies, animals were characterized by a decrease in live weight and the appearance of pathological changes in the lungs. In addition to these changes (weight loss and pathological changes in the lungs), our study has shown a decrease in body temperature and the death of unvaccinated hamsters. We did not find any information about these changes (a decrease in body temperature and death) in open sources on the Internet.

The results of pathomorphological, histological, molecular-genetic, and virological studies of the lung tissue showed that in all cases (both the control and experimental), virulent virus RNA was detected. When performing virus isolation in cell culture, the reproductive virus was detected from the lung tissue of unvaccinated hamsters challenged with the virulent virus, but was not detected from that of the vaccinated hamsters.

During the pathomorphological and histological examination, pronounced pathologies were found in the lungs of the control group of animals from the 3rd day, which persisted until the end of the experiment, as well as in the lungs of vaccinated animals, including insignificant changes noted on days 3–5, which completely disappeared by day 9.

The presence of viral RNA and the reproductive pathogen, accompanied by pronounced morphological destruction of lung tissue, indicate the development of acute respiratory disease in control animals as a result of infection with a virulent virus. The absence of a reproductive virus in the presence of pathogen RNA and minor morphological pathology in the lungs indicates the reproduction of a virulent virus in the surface epithelial cells of the respiratory system without penetration of the pathogen into the deep layers of tissue, where specific antibodies circulate, preventing the reproduction and spread of the pathogen. The reliability of this pathogenesis is confirmed by the results of tests of inactivated vaccines developed on the basis of related coronaviruses SARS-CoV and MERS-CoV (Roberts et al., 2010; Bolles et al., 2011; Tseng et al., 2012), as well as SARS-CoV-2 (Mohandas et al., 2021; Kandeil et al., 2021). Moreover, as in our studies, when an animal is infected with a virulent virus to which it was vaccinated, only a mild pathology develops in the lungs.

Investigating the pathogenesis of the disease caused by SARS and MERS viruses, a number of researchers observed the development of pathology in the lungs associated with antibody-dependent infection enhancement (Perlman and Dandekar, 2005; Jaume et al., 2012; Yip et al., 2014, 2016; Wang et al., 2016; Wan et al., 2020). In addition, there is also information that IgG-class antibodies to SARS-CoV S-protein antigens cause severe macrophage-mediated lung damage in both humans and great apes (Liu et al., 2019). Experiments on rabbits showed that animals re-infected with the MERS-CoV virus by the intranasal method developed pulmonary pathology, accompanied by viremia and severe lung inflammation, despite the presence of specific antibodies formed after the initial infection. In re-infected rabbits, lung damage was more severe than that during the primary infection (Liu et al., 2019). In other studies, when animals vaccinated against SARS-CoV (Tseng et al., 2012) or MERS-CoV (Agrawal et al., 2016) were infected with homologous virulent viruses, severe pneumonia developed, despite the high level of specific neutralizing antibodies in the vaccinated animals. Negative consequences from the use of the inactivated virus were also noted in other cases (Bolles et al., 2011).

Based on the above information, we paid close attention to the possibility of such a syndrome when using the QazCovid-in vaccine. However, the results of the studies did not confirm this probability, and the animals vaccinated with the test vaccine, with a control infection with a virulent virus, remained resistant to the disease without developing any visible clinical pathologies in their body. No signs of re-infection or antibody-dependent increased pathology were observed in our studies when animals vaccinated with QazCovid-in were infected with a virulent virus.

## Conclusion

The developed candidate vaccine QazCovid-in was shown to be safe with sufficient protective efficacy against COVID-19 caused by the SARS-CoV-2 virus in Syrian hamsters. The immune protection resulting from vaccination with the QazCovid-in vaccine suppressed the replication of the wild homologous virus in the body of vaccinated hamsters, reduced pneumonia, and ensured 100% animal survival. Based on the results obtained, the vaccine candidate has been approved for human trials and is currently in the final phase of phase III clinical trials in volunteers.

## Data Availability Statement

The datasets presented in this study can be found in online repositories. The names of the repository/repositories and accession number(s) can be found below: https://www.ncbi.nlm.nih.gov/nuccore/MZ379258.1.

## Ethics Statement

The animal study was reviewed and approved by Committee on the Ethics of Animal Experiments of the Research Institute for Biological safety problems of the Science Committee of the Ministry of Education and Science of the Republic of Kazakhstan (permit nos. KZ0520/013 and KZ1120/014).

## Author Contributions

KZh, KZ, LK, and MO: conceptualization. KS, ANr, ANk, SN, OC, BM, AI, NK, SK, AK, NA, YB, MM, MA, and MT: planning and conducting experiments. KZh, LK, KS, ANr, OC, and YB: data curation. KZh, LK, KS, ANr, OC, MO, BK, MK, and YA: formal analysis. KZ, YA, and MK: funding acquisition. YA and MK: investigation. KZh, LK, BK, MO, KS, and OC: methodology. KZ, LK, MO, and BK: supervision. KZh: writing–original draft. KZh, LK, MO, KS, OC, and BM: writing–review and editing. All authors have read and agreed to the published version of the manuscript.

## Conflict of Interest

The authors declare that the research was conducted in the absence of any commercial or financial relationships that could be construed as a potential conflict of interest.

## Publisher’s Note

All claims expressed in this article are solely those of the authors and do not necessarily represent those of their affiliated organizations, or those of the publisher, the editors and the reviewers. Any product that may be evaluated in this article, or claim that may be made by its manufacturer, is not guaranteed or endorsed by the publisher.

## Footnotes

1 Ventilation mode: 15 rpm, CO_2_ concentration: 0.04%, ammonia: ≤ 0.0001 mg/h, temperature: 19–22°C, humidity: 65–70%.
