# Supplementary material for: Development of the Inactivated QazCovid-in Vaccine: Protective Efficacy of the Vaccine in Syrian Hamsters
Source: Front Microbiol. 2021 Sep 27;12:720437. doi: 10.3389/fmicb.2021.720437 (PMC8503606; doi:10.3389/fmicb.2021.720437)
Supplement: Supplementary file 1 [file Data_Sheet_1.docx]

**Additional material 1 to the article “DEVELOPMENT OF THE INACTIVATED QAZCOVID-IN VACCINE: PROTECTIVE EFFICACY OF THE VACCINE IN SYRIAN HAMSTERS”**

**Isolation of the virus in cell culture from clinical samples**

Clinical samples. In this work, we used nosopharyngial flushes and whole blood obtained from 6 patients with the diagnosis of "SARS-CoV-2" undergoing therapy at the I. Zhekenova City Infectious Clinical Hospital in Almaty. These samples were taken by the medical staff of the city infectious diseases hospital and transferred to the Research Institute of Biological Safety Problems laboratory, following the international and domestic requirements for the transportation of clinical samples [1-3]. These samples were used as a source of the causative agent of coronavirus infection to infect the cell culture and then isolate this virus.

Cell cultures and culture medium. To isolate the virus, we used a Vero (WHO, Lot No. CB0 or CB884) transferable line, which was obtained from the WHO line. Vero cell culture was grown in DMEM (Invitrogen, Carlsbad, USA) with the addition of 2% veal serum and 200 mcg/ml of streptomycin, penicillin and 400 mcg/ml of gentamicin.

Infection and incubation of cell culture. After removal of the culture medium, blood and a suspension of nasal mucosa washes in the volume of 0.5 ml were applied to the monolayer of Vero cell cultures and kept for 60 minutes at a temperature of 37 °C. Then the inoculate was removed, the monolayer was washed in three shifts with PBS solution, DMEM maintenance medium was added with fetal blood serum, and cultivation was continued at

37 ^o^C with daily microscopy of the cell culture monolayer. The presence of the virus was determined by the cytopathogenic effect in the infected cell culture in comparison with the control uninfected cell culture. In the absence of CPE in a Vero cell culture infected with biomaterial samples, "blind" passaging was performed for at least three generations.

Virus titration. The infectious activity of the virus was determined by titration according to the Reed and Muench method [4] in a Vero cell culture grown in a 96-well culture microplate.In short, successive 10-fold dilutions of the virus source material from 10^-1^ to 10^-8^ were prepared in DMEM medium with the addition of 2% FBS, 100 u / ml of penicillin and 100 mcg / ml of streptomycin, and 100 mcl of the diluted virus was added to each well. The cells were incubated at 37 °C in an atmosphere of 5% CO**_2_** for 7 days, and the presence of a cytopathic effect was evaluated using an inverted microscope. The virus titer was calculated using the Reed and Muench formula and expressed in lg TCID_50_/ml

The studies were carried out in the laboratory base of the BSL-3 in accordance with the national requirements of biological safety for personnel and the environment.

**Isolation of SARS-CoV-2 virus in Vero cell culture.** Daily microscopy showed that from the third day after applying the smear-wash suspension of the nasal mucosa of one of the six patients, single rounded cells sharply refractive light (glowing) appear in the monolayer of Vero cell culture, and in the next two days the number of such cells increases sharply, which are massively rejected from the adhesion surface and float in the culture suspension in the form of balls without destroying their integrity. Over time, the number of rounded and desquamated cells increases (Fig. 1b), the number of flattened cells decreases on the adhesion surface (in the monolayer of the cell culture), and foci of "window-voids" form (Fig.1c) and intercellular spaces when compared with the control culture (Fig.1a). Complete destruction of the monolayer by exfoliation/desquamation of the affected cells occurred within 48-72 hours after the appearance of signs of cytopathology.


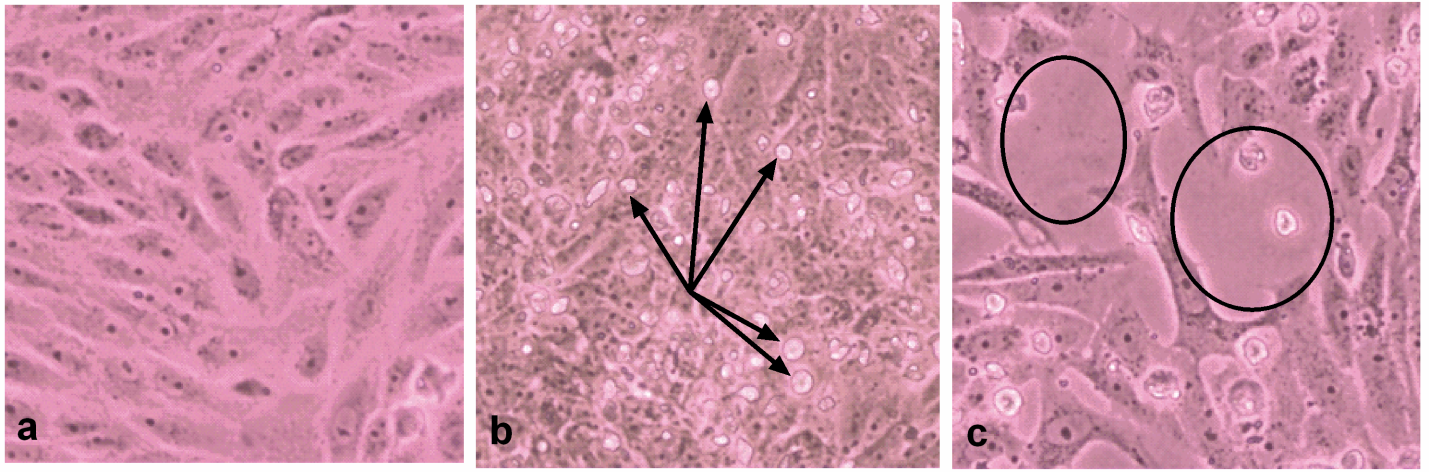


**Figure 1. a –** uninfected Vero cell culture, **b-**monolayer Vero cell culture infected with SARS-CoV-2 virus, 3 days after infection with the virus, **c-**monolayer Vero cell culture infected with SARS-CoV-2 virus, 4 days after infection with the virus. Black arrows indicate rounded and desquamable cells, and "empty windows" are indicated by a circle.

Passivation of the virus on a new batch of cell culture resulted in a more rapid manifestation of CPE. Cytopathogenic effect in Vero cell culture in the form of rounding, followed by the rejection of cells, initially single, and then massively, from the surface of adhesion from the third passage was noted already starting from the second day after infection. The character of the CPE remained the same as in the first passage. The titer of the 3rd passage virus in Vero cell culture was 10^4.83=0.27^ TCID_50/0.15 ml_.

It was not possible to isolate the SARS-CoV - 2 virus in the first three Vero passages from all blood samples and nasal mucosa washouts of the other 5 patients with COVID-19 coronavirus infection.

The negative results of virus isolation from whole blood and mucosal flushes of the remaining patients are probably due to the fact that there was no viremia in the body of the patients at the time of sampling and no virus excretion by excreta or the sensitivity threshold of the used cell culture.

**Conclusion.** For the first time in the Republic of Kazakhstan, the SARS-CoV-2 reproductive virus of the CoviD-19 coronavirus infection was isolated in vitro from a sick person, which has cytopathogenicity in Vero cell culture and has molecular genetic material identical to the pathogen isolated and identified in Wuhan, China.

The isolated virus isolate will be used for the monolecular-genetic certification and deposit of the pathogen in the republican bank / depository of microorganisms-pathogens of particularly dangerous infectious diseases, the study of its molecular-genetic and biological properties, as well as the development, standardization, production and introduction into wide practice of means and methods of laboratory diagnostics and specific prevention of the disease.

**References**

1. WHO/WHE/CPI/2019.20 Guidance on regulations for the transport of infectious substances 2019– 2020
2. OIE Terrestrial Manual. 2018. Transport of biological materials. Chapter 1.1.3.
3. Order of the Minister of Health of the Republic of Kazakhstan dated September 8, 2017 No. 684 "On Approval of Sanitary Rules" Sanitary and epidemiological requirements for laboratories using potentially dangerous chemical and biological substances»
4. Reed, L. J. & Muench, H. A Simple Method Of Estimating Fifty Per Cent Endpoints12. *American Journal of Epidemiology* **27**, 493–497,

<https://doi.org/10.1093/oxfordjournals.aje.a118408>(1938).
